# Characterization and evaluation of cold atmospheric plasma as seedborne fungal disinfectant and promoting mediator for physico-chemical characteristics of *Moringa oleifera* seedlings

**DOI:** 10.1038/s41598-022-18768-7

**Published:** 2022-09-22

**Authors:** Salama A. Ouf, Jamal Q. M. Almarashi, Abdel-Aleam H. Mohamed

**Affiliations:** 1 Botany and Microbiology Department, Faculty of Science, Cairo University, Giza, 12613 Egypt; 2 Physics Department, Faculty of Science, Taibah University, Madinah, 42353 Saudi Arabia; 3 Physics Department, Faculty of Science, Beni-Suef University, Beni-Suef, 52511 Egypt

**Keywords:** Biophysical methods, Microbiology techniques

## Abstract

Non-thermal atmospheric pressure plasmas are a powerful tool to impact seed germination and microbial decontamination. Air large volume atmospheric pressure glow discharge plasma was developed and investigated to improve the biological activities of *Moringa oleifera* seeds. Ninty ns magnetic pulse compression high voltage system was used to generate the plasma. The plasma discharges current increases with increasing applied voltage and it decreases with increasing discharge gap. There was a steady reduction in the count of seedborne fungi on the application of air cold plasma with complete elimination of fungi at ≥ 10.94 mJ per pulse. The low doses of plasma (2.46 and 4.35 mJ) induced an increase in the seed germination, a significant increase in chlorophyll content (chl *a* and chl *b*) and antioxidant activities of the seedlings emerged from soaked or wet seeds rather than dry seeds. At lower plasma doses (2.46 and 4.35 mJ) there was a significant increase in leaf area and chlorophyll content (chl *a* and chl *b*) of the seedlings that emerged from H_2_O_2_ soaked seeds rather than that of free from H_2_O_2_. The plasma was harmful when applied at higher doses (≥ 10.94 mJ) and more harmful to the wet seeds compared to the dry ones.

## Introduction

*Moringa* is related to a family Moringaceae and is called a miracle tree as all part of the plant is used for food. The seeds of the plant are employed in water purification, the leaves as food additions, the oil as biodiesel, the trunks as gum, the flowers as honey, and all parts of the plant can also be used for pharmaceutical purposes^1^. *M. oleifera,* which is also known as the “Miracle Tree” and “Mother's Best Friend,” is considered the most nutrient-rich plant. Other than having a high concentration of vitamin A, vitamin C, potassium, and calcium, the plant contains all the essential amino acids^2^. Moreover, *Moringa* species contain various phytochemical content such as alkaloids, saponins, tannins, steroids, phenolic acids, fatty acids, glucosinolates, flavonoids, and terpenes. The variety of these constituents contributes to its several pharmacological uses^3^. Moringa species were reported to have a wide spectrum of biological impacts such as antioxidant activities^4^, anticonvulsant^5^, anticancer^6^, antibacterial^7–9^, antifungal^10^, antiviral^11^, tissue sterilization from skin flora in seconds, and blood clot formation^12^, cancer therapy^13^, antifertility^14^, and promoting apoptotic behavior in melanoma skin cancer cell lines^15^.

Although the bulk of these traditional methods is concerned with stimulating germination seedling establishment and growth of plants, the procedures are time-consuming, expensive, sometimes cause seed damage, have low efficiency, and may be environmentally pollutant. Previous studies of Ouf and Abdel-Hady^16^ working with the radiation, indicated that He–Ne laser exposure of soybean seeds may induce an increase or decrease in growth, nodulation, chlorophyll, and carotenoid contents as well as the resistance of the seedling to *Fusarium solani*, depending on the irradiation dose and the applied photosensitizer to the seeds.

It was known that ionizing radiation causes physicochemical steps in the biological system including the preliminary excitation and ionization of water molecules into free radicals leading to a chain of reactions that produce secondary free radicals. The efficacy of cold atmospheric plasma in the proposed applications may rely on the synergistic action of the reactive oxygen species (ROS), reactive nitrogen species (RNS), free radicals, UV photons, charged particles, and electric fields^17,18^. Cold plasma contains electric field, ozone, atomic oxygen electrons, ions, radicals, and other active species, like excited atoms and molecules, which are being used in the modification of temperature-sensitive materials like biological cells, microorganism sterilization, and improvement of seed performance and crop yield. Due to the unique features of plasma, it is known as a fast, uniform, economic, nondestructive, effective, and ecologically safe treatment method.

The generation of multi-jet plasma has been investigated previously. Weltmann and co-workers are considered to be a pioneer in this point of research by generating several Rf argon plasma jets^19^. M. G. Kong team has a significant contribution to generating helium AC (30 kHz) plasma array and they studied the interactions between jets^20,21^. The possibility of generating multi atmospheric pressure plasma jets through individual ballast and the strong interplay between the discharge and the gas flow and the interaction of multiple plasma plumes were investigated^22–24^. Microsecond pulsed driven multi-jet were developed and the electric field time-resolved was measured as well as the voltage polarity effect was investigated for helium and neon atmospheric plasm jet arrays^25,26^. The previous work of multi-jet arries is operated using rare gases such helium, argon, or neon while in this work the large atmospheric pressure plasma is operated in the air between two metallic electrodes.

The atmospheric pressure plasmas were characterized electrically and spectroscopically. The electric field distribution was investigated and measured using nonintrusive and nonperturbative methods^27^. These studies indicated that both the axial and radial electric field components have an amplitude of several kV/cm. In previous work, a simulation to study the propagation of air discharges at atmospheric pressure in dielectric tubes was carried out^28^.

It was established that seed treatment with plasma results in favorite morphological and biochemical characteristics through the surface and internal modification by the charged or neutral species designed in the plasma^29^. Such species can interact with the seed surface and partially pierce into the seed, thus motivating the biochemical progressions mandatory for seed germination.

*Moringa oleifera* is one of the world’s most important promising plants. Due to the poor and delay in the germination of *M. oleifera*, it was found that most seedlings fail to establish and continue growth probably due to microbial contamination resulting from the associated seedborne and soilborne fungi that cause rotting or damping-off of the seed. The aim of the present investigation was the characterization and optimization of cold plasma for fungal disinfection of the seeds as well as improvement of seedling characteristics particularly chlorophyll and antioxidants by atmospheric pressure cold plasma treatment.

## Results and discussion

### Large volume air plasma characteristics

**Figure 1:**
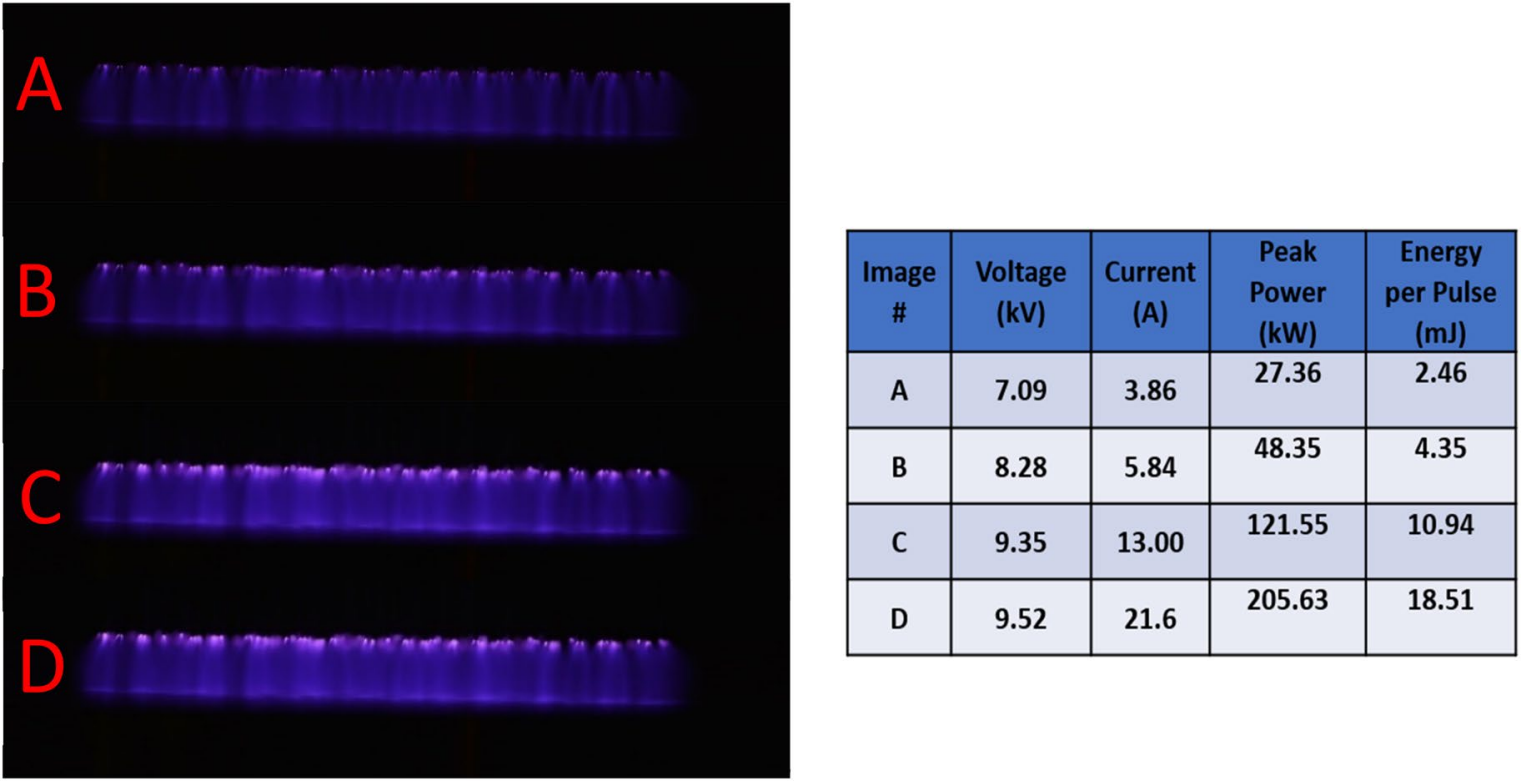
(**A**–**D**) Images of the large volume air plasma formation as a function of increasing energy per pulse at 2000 Hz. On the right, the table presents the corresponding applied voltage and discharge current, the peak power and the energy per pulse for each image.

The atmospheric pressure plasma formation was operated at 2000 Hz and 15 mm gap, as a function of increasing the input energy to the pulsed power supply system. As the input energy increases the plasma light emission intensity increase due to the increase in discharge current and applied voltage as shown in Fig. 1. Small glows are formed on the pin tips at the initiation of the glow discharge when the lower applied voltage is established between the two electrodes. The glow discharges are generated at the outer pins initially and its luminousness is intense and spread to cover the discharge gap laterally and axially with increasing the applied voltage (Fig. 1A–D). As well as the plasma gets diffuse and individual plasma columns merge by increasing the applied frequency and applied voltage^30^. The merging between individual plasma columns has been reported previously for a micro-hollow cathode sustained direct current air glow discharge^31^.

**Figure 2:**
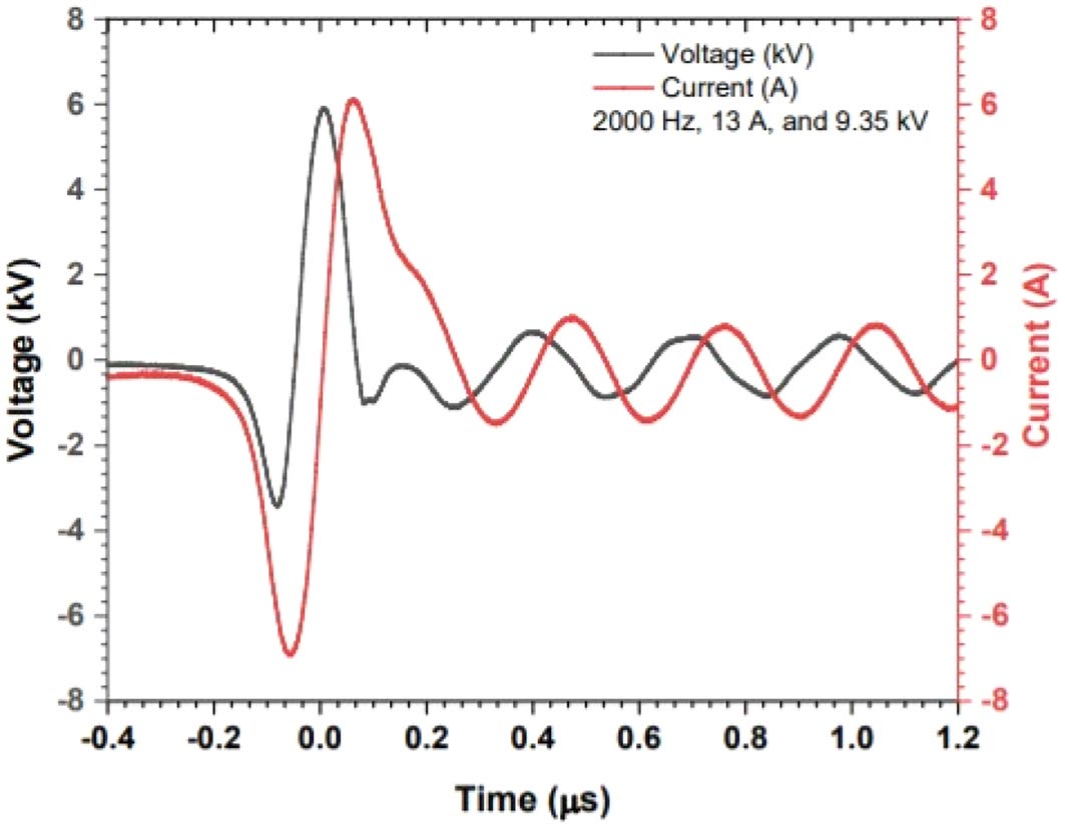
The typical current–voltage waveforms for the large volume atmospheric pressure air plasma at operating conditions of 2000 Hz and 1.5 cm discharge gap 2000 Hz, 13 A, 9.35 kV and 10.94 mJ.

Figure 2 shows a typical current–voltage waveform for the large volume atmospheric pressure air plasma that operated at 2000 Hz applied frequency, 15 mm discharge gap, 10.94 mJ energy per pulse, and 9.35 kV applied voltage. 90 ns was the estimated pulse duration of the positive half of the applied voltage waveform. The applied voltage and the discharge current measured the difference between the first (negative) and first (positive) peaks of the voltage and current waveforms. The discharge current increases with increasing the applied voltage, as the applied voltage increased from 7.09 to 9.52 kV, the discharge current increased from 3.86 A to 21.6 A. The peak power was measured by multiplying the voltage by the current and the energy per pulse was obtained by multiplying the power by the pulse duration (90 ns). An arc discharge is formed when a further increase in the applied voltage is established. The discharge current variation with increasing the applied voltage indicates that the plasma is operating in the abnormal glow mode and resembling the point-to-plane negative corona discharges^32^. The operation of the large volume discharge plasma was limited below the glow to arc transition which depends on discharge gap distance, applied voltage, and frequency.

**Figure 3:**
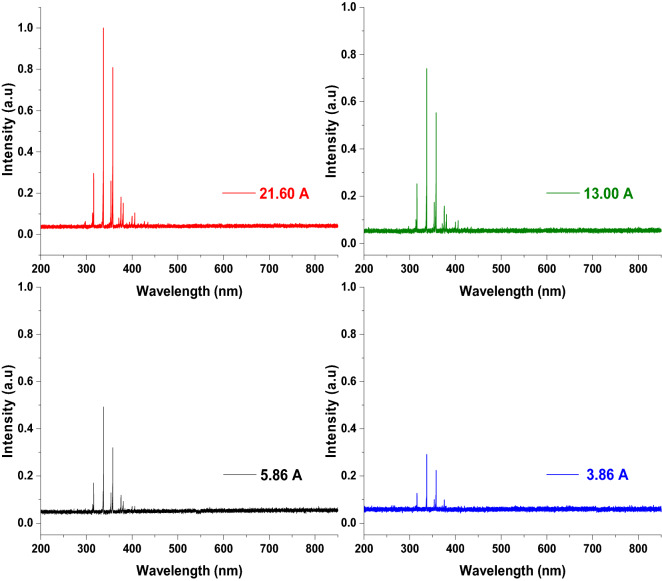
Emission spectra of large volume air plasma at different discharge currents and fixed operating conditions of 2000 Hz and 15 mm discharge gap.

The optical emission spectra are an indication of the plasma contents. The measured emission spectra from large volume atmospheric pressure plasma, at different discharge currents of 21.60, 13.00, 5.84, and 3.86 A and fixed operating conditions of 2000 Hz discharge frequency and 1.5 cm discharge gap, indicated the presence of nitrogen molecule bands. The spectra show the presence of the first negative and second positive systems of the nitrogen molecule and the second positive system emission intensity was the highest in the investigated range between 200 and 800 nm (Fig. 3). The emission spectra from NO, OH, and O radicals were not detected. The intensity of most of the detected nitrogen bands emission spectra increases with increasing discharge current.

**Figure 4:**
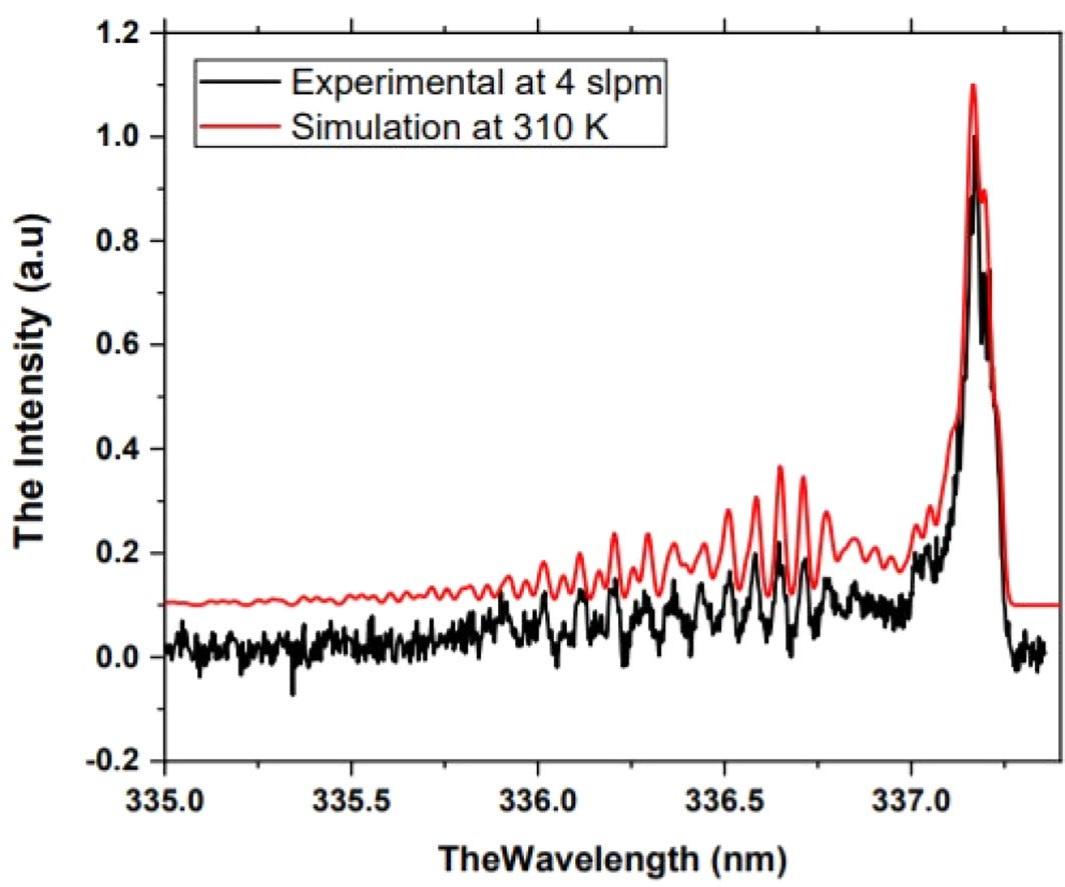
Atmospheric pressure large volume air plasma gas temperature measurement: the estimated gas temperature is 310 k at operating conditions of 2000 Hz and 15 mm discharge gap 2000 Hz, 13 A, 9.35 kV, 10.94 mJ.

The large volume of atmospheric pressure air plasma gas temperature was measured side-on at the middle of the 15 mm discharge gap using an optical fiber bundle at the center of one of the plasma columns. The gas temperature was measured by evaluating (0, 0) transition of the N_2_ second positive system rotational band. The gas temperature was determined by the best match between the measured 0–0 transition of the second positive system of nitrogen spectrum (C3Πu → B3Π) and the simulated one. Figure 4 presents the results of the estimated gas temperature of the generated plasma which was operated at operating conditions of 2000 Hz and 15 mm discharge gap 2000 Hz, 13 A, 9.35 kV, 10.94 mJ. The results show that the gas temperature was in the range of 310 ± 20 K. The results show that the generated plasma is an air non-thermal plasma operated at atmospheric pressure^31,32^.

### Seedborne fungi

Several investigators indicated that cold plasmas have several mechanisms of action and can inhibit different microorganisms contaminating food and agricultural products^33,34^. Their action includes the inactivation involving oxidative DNA damage and membrane lipid peroxidation^35^.

In this research, forty-one fungal colonies related to 13 species were recovered from *M. oleifera* dry seeds, about one-third of which were related to *Aspergillus* species (Table 1). *A. flavus* and *A. niger* were the dominant according to the quantitative occurrences. *Cladosporum herbarum* and *Penicillium chrysogenum* each were recovered 5 times from the investigated seeds. *Rhizopus* spp. were isolated in 6 colonies/10 g seeds. Each of *Alternaria humicola* and *Fusarium oxysporum* was represented with 3 colonies. The other species have emerged in one or two colonies. On water or H_2_O_2_ soaking, 4 and 10 colonies were missed, respectively. There was a steady reduction in the total count of emerged fungi to 11 colonies and 2 colony/10 g seeds on the treatment of the seed soaked in water with 2.46 mJ and 4.35 mJ of energy per pulse, respectively. With the same doses, only 8 and one colony/10 g were recovered from H_2_O_2_-soaked seeds, respectively. The higher doses (≥ 10.94 mJ) induced a complete elimination of seedborne fungi. Seeds of different plants are commonly associated or within seeds with fungal pathogens causing rotting, wilting, or damping off. These fungi may remain inactive mycelial hyphae or spores within the seeds and cause new infections and disease dissemination to a new location^36^. El-mohamedy et al.^37^ stated that the poor germination and low establishment of *M. oleifera* seedlings were related to the susceptibility of seeds to the soil fungi. Mitra et al.^38^ indicated that the cold plasma treatment inactivates the seedborne microbial population, consequently avoiding health risks and economic loss related to microbial contamination.

**Table 1:** Effect of cold atmospheric plasma on seedborne fungi associated with *Moringa oleifera* (colony/10 g dry seeds).

Fungal species	Untreated seeds			Treatment					
	Dry seeds (control)	Water-soaked seeds	1% H_2_O_2_ soaked seeds	Seeds treated with 2.46 mJ		Seeds treated with 4.35 mJ		Seeds treated with 10.94 mJ	
				A*	B*	A	B	A	B
*Asperigillis flavus*	6	4	3	2	1	0	0	0	0
*A. candidus*	1	1	1	0	0	0	0	0	0
*A. terreus*	2	2	2	1	1	0	0	0	0
*Asperigillis niger*	6	5	4	2	2	1	1	0	0
*Alternaria humicola*	3	3	3	0	0	0	0	0	0
*Chaetomium globosum*	1	1	1	0	0	0	0	0	0
*Fusarium oxysporum*	3	3	2	2	2	0	0	0	0
*F. solani*	1	1	0	0	0	0	0	0	0
*Rhizopus oryza*	4	3	4	1	0	0	0	0	0
*R. nigricans*	2	2	1	0	0	0	0	0	0
*Cladosporum herbarum*	5	4	4	1	1	0	0	0	0
*Macrophomina phaseolina*	2	2	2	0	0	0	0	0	0
*Penicillium chrysogeum*	5	5	4	2	1	1	0	0	0
Total count	41	37	31	11	8	2	1	0	0

*A = Seeds soaked in water, B = seeds soaked in 1% H_2_O_2_.

### Seed germination

**Figure 5:**
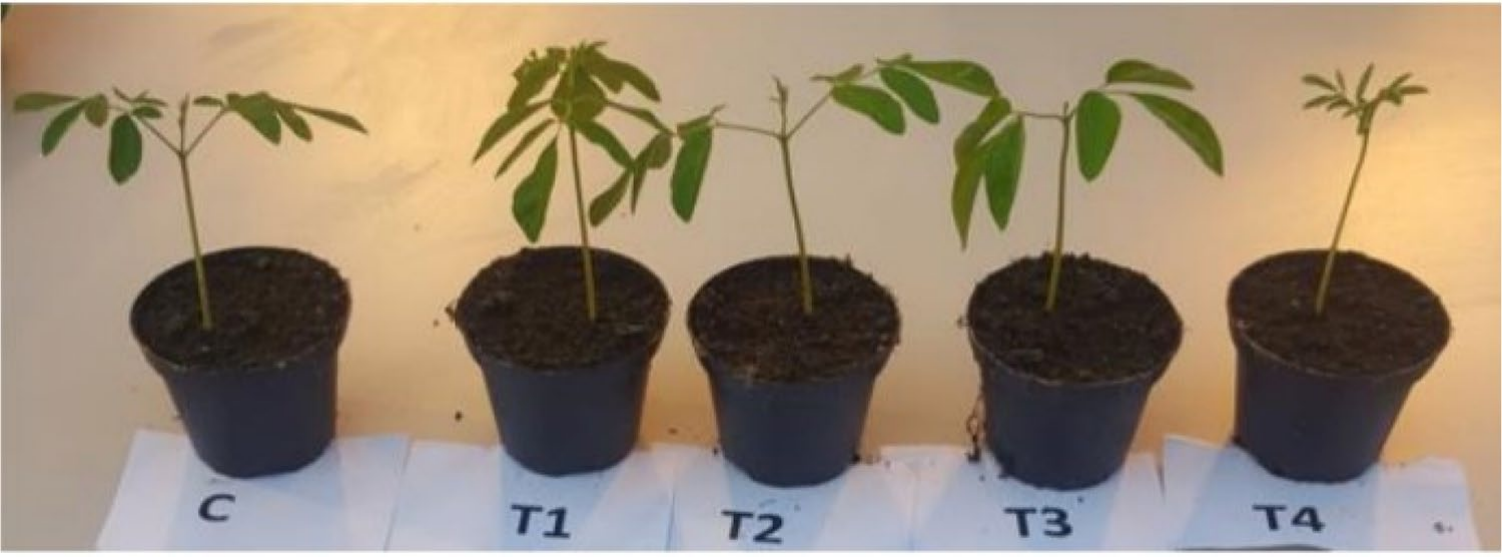
Seedlings of *M. oleifera* emerged from H_2_O_2_ soaked seeds treated with atmospheric pressure cold plasma for 10 min at doses of 2.46 mJ (T1), 4.35 mJ (T2), 10.94 mJ (T3), and 18.51 mJ (T4) compared to seedlings emerged from untreated non-H_2_O_2_ soaked seed (C).

The low doses of cold plasma (2.46 and 4.35 mJ) induced an increase in the seed germination of *M. oleiferea* which was more obvious and significant in the case of H_2_O_2_ soaked seeds. On the other hand, seed treatment with higher doses (10.94 and 18.51 mJ) induced marked inhibition in seed germination which was more pronounced in seedlings that emerged from H_2_O_2_ soaked seeds reaching 72.6 and 38.4% in the case of treatment with 10.94 and 18.51 mJ, respectively (Table 2). The results recorded for the germination rate match with those recorded for germination. The maximum leaf area was estimated for the seedling developed from 2.46 mJ of cold plasma energy per pulse in the case of H_2_O_2_ soaked seeds followed by seeds soaked in H_2_O_2_ free water (Fig. 5). The higher doses (10.94 and 18.51 mJ) significantly reduced leaf area compared with the control, though the reduction was more obvious with H_2_O_2_-soaked seeds.

**Table 2:** Percentage germination and average leaf area of *Moringa oleifera* seeds treated with different doses of atmospheric pressure cold plasma for 10 min.

Dose of cold plasma (mJ)	Water-soaked seed			1% H_2_O_2_ soaked seed		
	% Germination	Germination rate	Leaf area (cm^2^)	% Germination	Germination rate	Leaf area (cm^2^)
0 (control)	81.3 ± 3.2	8.0 ± 0.5	1.126 ± 0.092	81.3 ± 3.2	8.0 ± 0.7	1.120 ± 0.082
2.46	86.4 ± 2.9	9.6 ± 0.5	1.237 ± 0.083	83.5 ± 2.7	9.3 ± 0.6	1.175 ± 0.091
4.35	93.1 ± 3.0	8.8 ± 0.4	1.529 ± 0.081	90.8 ± 3.0	8.5 ± 0.6	1.398 ± 0.075
10.94	72.6 ± 2.8	6.8 ± 0.6	0.742 ± 0.88	77.4 ± 2.8	7.5 ± 0.5	0.825 ± 0.068
18.51	38.4 ± 2.9	5.5 ± 0.4	0.604 ± 0.91	43.8 ± 2.2	6.0 ± 0.6	0.732 ± 0.078

Although several investigations were carried out on the effects of plasma on seeds, however, the motivating activity of cold plasma as a mediator in the enhancement of seeds germination and seedling growth is not fully clear. It is thought that the facility of water uptake and the changes occurring on the external seed surface enhance the hydrophilic ability of the seed and increase mobilization and solubilization of the reserve food in the seeds and interactions of cellular components, so initiating the growth and development of the seedlings^39^. The application of cold plasma may alter the seed surface wettability leading to more water absorption^40^.

The reactive species emitted from cold plasma may cause breaking of the seed dormancy^41^ or induce scratching, clefts, or erosion in the seed coat due to interaction with the seed surface, so facilitating water uptake^18^. Dawood indicated that treatment of *M. oleifera* seeds by cold plasma (RF-Ar low-pressure plasma) for one and five minutes improved the germination parameters as well as root and shoot potentials^42^. The addition of H_2_O_2_ to the soaking water increases the efficiency of the cold plasma through the increased liberated active species that may contribute to plant increment in growth and development. Hydrogen peroxide molecule initiates the production of other diverse reactive oxygen radicals, such as superoxide, hydroxyl, and NO_x_, in cells. H_2_O_2_ primarily acts as a vital signaling molecule and stimulates the production of other signaling molecules such as enzymes, hormones, jasmonic acid, abscisic acid, and ethylene^43^.

Szili et al.^44^ suggested a strategy employing cold plasma for the "on-demand" activation of acetyl donor molecules. The process generates an aqueous-based antimicrobial formulation comprising a rich mixture of highly oxidizing molecules: peracetic acid, hydrogen peroxide, and other reactive oxygen and nitrogen species. The synergistic powerful oxidative action between these molecules is shown to be highly effective at eradicating microbial seed pathogens.

According to the literature review, the application of the cold plasma technique does not cause any change or mutation in the genetic material of seeds^45^. However, Šerá et al. indicated that the active species of cold plasmas could pierce through the seed coats and excite natural signals such as growth factors^46^. This situation may induce regulation of the demethylation levels of certain genes, which leads to the promotion of germination and seedling growth^47^. Sidik et al. working with corn plants showed that the seeds that were treated with 3 min of cold plasma germinate faster and show a better growth rate related to the control seed^48^. This revealed that cold plasma treatment is a suitable and standard technique to enhance seed germination and promote seedling growth of the plant.

### Chlorophyll content

The chlorophyll pigment contents of the seedlings grown from cold plasma-treated seeds differ significantly according to whether the seeds were soaked in water or 1% H_2_O_2_ solution. At lower plasma doses (2.46 and 4.35 mJ) there was a significant increase in chlorophyll content (chl *a* and chl *b*) of the seedlings that emerged from H_2_O_2_ soaked seeds rather than that free from H_2_O_2_. At higher doses of 10.94 mJ and 18.51 mJ, the plasma was harmful, particularly when applied to H_2_O_2_ soaked water, where the chlorophyll *a* content of the seedling measured 6.1 ± 0.4 and 5.2 ± 0.4 mg/100 g for seedling developed from seed soaked in 1% H_2_O_2_ compared to 7.3 and 6.3 mg/100 g for seedlings appeared from seeds soaked in H_2_O_2_-free water, respectively (Table 3).

**Table 3:** Effect of atmospheric pressure cold plasma applied for 10 min to *M. oleifera* dry or soaked seeds on chlorophyll content of seedling after 30 days emerged from water soaked or H_2_O_2_ soaked seeds.

Dose of cold plasma (mJ)	Chlorophyll content (mg/100 g seedling leaf materials)			
	Water-soaked seeds		H_2_O_2_ soaked seeds	
	Chl *a*	Chl *b*	Chl *a*	Chl *b*
0 (control)	6.7 ± 0.3	5.3 ± 0.3	6.9 ± 0.4	5.4 ± 0.5
2.46	7.3 ± 0.4	6.2 ± 0.5	7.6 ± 0.4	6.4 ± 0.4
4.35	7.9 ± 0.4	6.6 ± o.4	8.6 ± 0.3	6.9 ± 0.4
10.94	7.3 ± 0.5	5.6 ± 0.4	6.1 ± 0.4	4.4 ± 0.3
18.51	6.3 ± 0.3	4.3 ± 0.5	5.2 ± 0.4	3.7 ± 0.5

High levels of chlorophyll in plasma-treated seeds can be attributed to the increase of physiological activity and photosynthesis in plants. Saberi et al. reported that plasma treatment of winter wheat (Pishgam cultivar) for 180 s improved photosynthesis rate, chlorophyll content, and stomatal conductance by 34, 32, and 93%, respectively, compared with the control^49^. However, other researchers have emphasized the positive effects of plasma on increasing chlorophyll content in tomatoes and maize^50,51^. Šerá et al. reported no significant changes in chlorophyll content in plasma treatment on rape seedlings^45^. Jiafeng et al. in their field experiments with wheat seeds treated with 80 W cold plasma, indicated that the chlorophyll content increased by 9.8% higher than those of the control, indicating that cold plasma treatment could promote the growth and the yield of treated plants^44^.

### Antioxidant activities

**Figure 6:**
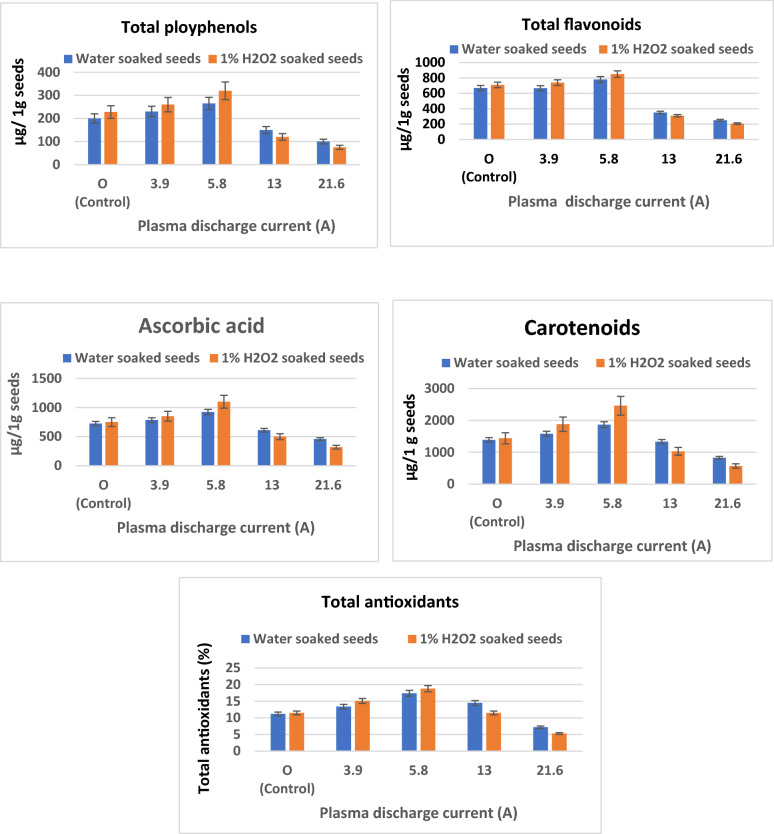
Effect of cold plasma discharge current applied for 10 min to *M. oleifera* soaked seeds on seedling antioxidants after 30 days of growth.

The antioxidant activity of *M. oleifera* seeds was evaluated in 30-day developed seedlings after exposure of the seeds to differing doses of large volume plasma for 10 min. The lower doses (2.46 and 4.35 mJ) are simulative for the antioxidant activity of seedlings, particularly those that emerged from plasma-treated H_2_O_2_ pretreated seeds reaching 18.8% compared to 11.5% in the case of the corresponding untreated seeds. The same trend was observed in the case of assessing total polyphenols, total flavonoids, ascorbic acid, and carotenoids reaching 320, 850, 1100, and 2460 µg/1 g seed compared to 228, 710, 750, and 1440 µg/1 g for the seedlings that emerged from untreated seeds under the same conditions, respectively (Fig. 6). Ling et al. indicated that the treatment of oilseed rape (*Brassica napus* L.) with cold plasma evidently increased the level of superoxide dismutase and catalase activities by 13.00–17.71% and 13.21–16.52%, respectively^52^. Moreover, cold plasma treatment significantly induced an increase in the soluble sugar and protein contents suggesting that cold plasma treatment improve the drought resistance of the plant through the improvement of antioxidant enzyme activities, increasing osmotic-adjustment products, and reducing lipid peroxidation.

It has been reported that the low doses of cold plasma enhance the diversity of metabolic and physiological activities including antioxidant capacity, while it causes oxidative stress in seeds at higher doses^53^. Under such stress, plants are well equipped with a fundamental antioxidant defense system comprising enzymatic antioxidants, as well as non-enzymatic antioxidants, such as ascorbate to resist oxidative stress^54^. These antioxidants are important for motivating physiological and developmental processes and resisting stresses^55^.

The higher plasma dose (> 18.51 mJ) induces inhibition in total antioxidants compared to smaller doses although the value is still around the control value. However, total polyphenols, total flavonoids, ascorbic acid, and carotenoids were markedly decreased recording 120, 310, 500 and 1030 µg/1 g for H_2_O_2_ soaked seeds exposed to 10.94 mJ for 10 min compared to the values recorded for control. A higher dose (> 18.51 mJ) induced remarkable inhibition for seedlings that emerged from plasma-treated seeds and was more evident for seedlings developed from H_2_O_2_-soaked seeds. At higher doses, more reactive ionic species are liberated, and the overproduction of these species may lead to oxidative stress damage to DNA, proteins, and lipids^56^.

## Conclusion

Plasma technologies have been demonstrated as promising and potential technology in many different aspects such as microbial decontamination and improvement of plant characteristics. Cold plasma is suitable for seed treatment as it does not leave residual pollution affecting or interrupting the function of tissues and provides uniform treatments. Moreover, the use of non-thermal plasma in improvement seed germination and growth characteristic may be useful in the production of crop yield and cause significant biochemical reactions inside the seeds, and eliminate the microbial decontamination of the seeds, suggesting that it may provide an alternative to traditional or chemical promotors or pesticides. The reactive species in plasma interact with the seed surface or penetrate the seed, thereby stimulating biochemical processes required for seed germination and inhibiting the associated pathogenic microorganisms. The successful seed decontamination treatment possibly will cause the inactivation of associated microbial pathogens and preserves seed viability, germination, and vigor. Several applications of plasma in agriculture have recently been described, including the proposed application of plasma-activated water and soil sterilization. Therefore, plasma can be used not only for the treatment of seeds but also during the entire field cultivation period.

## Materials and methods

### Plasma system and its characteristics

**Figure 7:**
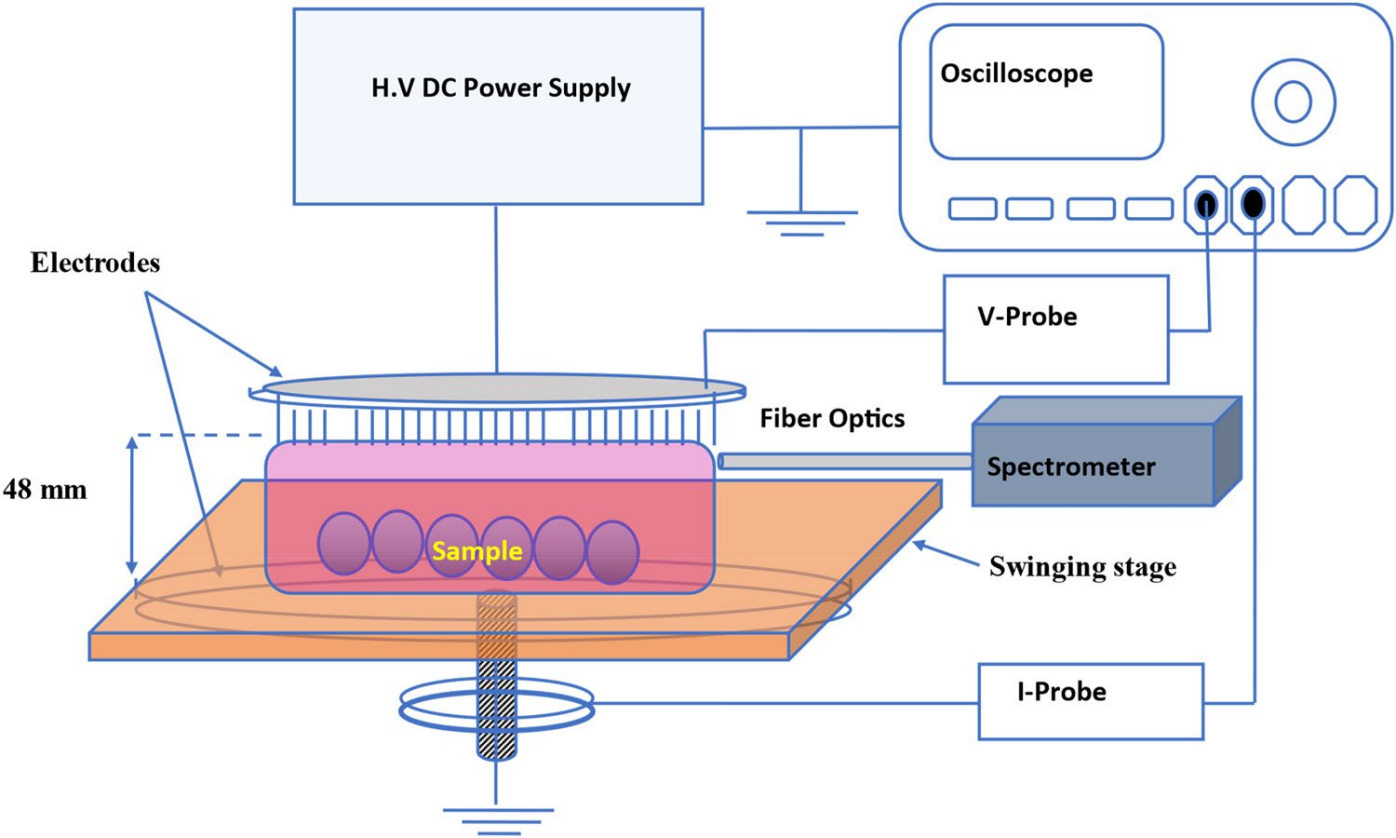
Large volume atmospheric pressure air plasma experimental setup system.

A high voltage magnetic pulse compression power supply was used to generate large volume atmospheric pressure air plasma when applying 100 ns high-voltage pulse between two parallel electrodes. The plasma system parameters can be varied as follows; from 1 to 2000 Hz applied frequency, 0–18.51 J energy per pulse, and from 0 to 48 mm discharge gap. The power supply used in this system is a high-voltage pulse generator based on magnetic pulse compression (MPC) and it is called "HYGEIA" that has a commercial name of «Proteus-II», Electrodynamic systems & technologies LLC, Greifswald, Germany. The upper electrode has uniformly distributed stainless-steel pins with 28 mm length and has ~ 0.1 mm diameter for each pin that is separated by a 10 mm distance from each other to form a brush shape with 160 mm diameter. The upper electrode is connected to the high voltage power supply while the lower electrode is grounded and has a flat disc shape. An automated movable stage with a flat stainless-steel surface 180 $\times$ 180 mm^2^ which is swinging by 15 mm at 0.3 Hz was placed on and in contact with the ground electrode. The discharge gap is measured from the tip of the pins to the surface of the movable stage of the grounded electrode. The desired treated samples were placed on the flat surface of the movable stage that swinging to increase the homogeneity of the plasma treatment. DPO7354 C − 3.5 GHz-Tektronix oscilloscope was used to record the current and voltage pulses to investigate the electric characteristics of the generated plasma. A calibrated high voltage probe and a 1:1 Pearson current probe, model: 6585 were used to measure the current pulses. The voltage pulses were measured using 1:1000, P6015A-Tektronix probe. The current pulses were measured through the grounded electrode and the applied voltage pulses were measured across the discharge gap as illustrated in Fig. 7. Nikon digital camera D3200 with AF-S Micro NIKKOR 105 mm lens was used to record and study the plasma formation. Pictures of the generated large volume air plasma at different input energy and its corresponding applied voltage and discharge current is presented in Fig. 1.

The large volume atmospheric pressure air plasma emission spectra were investigated using triple grating Acton SP-2356 UV–VIS-500 mm focal length spectrograph that is coupled by LG-455-020-3 single-leg-fiber optics bundle. The spectrograph grating used in this work is 1800 g/mm that blazed at 500 nm. Princeton instrument model ARC-P2 highly sensitive photomultiplier is used as a detector with the spectrograph which has a sensitivity range from 200 to 900 nm. The atmospheric pressure plasmas collision frequency is very high. Consequently, a fast thermal equilibrium between the rotational and translational energies is established very fast (~ 10^–6^ s). Therefore, for atmospheric pressure plasmas, the rotational temperature can be considered as the gas temperature. In this work, the generated air plasma gas temperature was considered as the rotational temperature that was estimated from the emission spectra of the (0–0) transition of the second positive system of nitrogen molecules spectra, [C_3_Π_u_ → B_3_Π_g_].

#### Tested seeds

*Moringa oleifera* seeds were obtained from Horticultural Research Institute, Agriculture Research Center, Giza, Egypt. Experimental research and field studies on the tested plant or plant material (flowers, seeds, leaves, fruits, etc.), have comply with relevant institutional, national, and international guidelines and legislation. The seeds were immediately transferred to the laboratory where they were treated with cold plasma before being tested for germination, fungal contamination, and other further biochemical analyses.

#### Cold plasma

The cold plasma was applied to the seeds for 10 min at 2.46, 4.35, 10.94, and 18.51 mJ of energy per pulse where the pulse duration was 90 ns. The corresponding applied voltage and discharge current is presented in Fig. 1. Preliminary experiments showed that the seeds were not significantly affected when the plasma was tried at lower doses $<$ 3.9 A and fall to grow at doses $>$ 21.6 A.

#### Seed germination and seedling growth

The experiment was conducted in the greenhouse for two successive seasons during July 2018 and 2019 in the agriculture farm of Agriculture Research center, Ciza, Egypt. Seeds of *M. oleifera* were soaked in tap water or tap water with 1% H_2_O_2_ for 12 h. The soaked seeds were subjected to cold plasma at the investigated dose. The experiment was carried out using 15 cm diameter pots filled with sand compost mixture in 2:1 ratio. One seed was cultivated in each a series of 10 replicate pots of each treatment. Untreated seeds were used as a control. The experiment was continued for 28 days. The percent of emerged seedlings was recorded by the end of the experiment. The germination percentage, rate of growth, average leaf area, and other investigated parameters were investigated under different plasma doses. The experiment was organized in a randomized complete block design.

### Isolation of seedborne fungi

The dilution plate method as described by Johnson et al.^57^ was adopted for the isolation and counting of fungi. Ten-gram dry seeds were aseptically transferred to 250 ml conical flask containing 90 ml sterilized distilled water and shaken for 15 min. Czapek–Dox's agar media was used as an isolation medium. A drop of streptomycin was added to each plate for the suppression of bacterial growth. Ten replicate plates were used for each treatment. The emerging colonies were incubated for 5–10 days at 28 °C and estimated per gram dry material and identified by microscopic and physiological examination through the help of the manuals of Barnett and Hunter for imperfect fungi^58^, Barron for the genera of hyphomycetes^59^, Ellis, for soil fungi^60^, Kendrick for imperfect fungi^61^, Moubasher for soil fungi in Qatar and other Arab countries^62^ and Raper and Fenell for *Aspergillus* genus^63^.

### Leaf chlorophyll contents

Fresh leaves developed from untreated or treated seeds were cleaned to get rid of adhered soil particles and other contaminants. Leaves collected from 28-day-old seedlings developed from treated or untreated seeds were collected. Leaf chlorophyll was extracted using 80% acetone according to following Xue’s technique^64^. Chlorophyll *a* and chlorophyll *b* were measured at wavelengths 645 and 655 nm, β-carotene, and lutein at wavelengths 480 and 495 nm. Each sample measurement was performed in three replicates and the pigment content was calculated from equations presented by Bulda et al.^65^. The absorbance value of the clarified chlorophyll extracts was measured using a UV–visible spectrophotometer (Pharma Spec, UV-1700, Shimadzu, Japan) at 645 and 663 nm, and the chlorophyll *a* (Chl-*a*), chlorophyll *b* (Chl-*b*), were calculated. Leaf chlorophyll contents were determined with an average of three replicates.

### Determination of antioxidant activity of samples

Five grams of *M. oleifera* seedlings were extracted by 100 ml, 80% methanol. The total antioxidant activity of seedling samples was estimated by the DPPH (Diphenyl-1picrylhydrazyl) radical-scavenging method based on the ability of antioxidants to block the 2,2-Diphenyl-1picrylhydrazyl radical^66^. The sample was incubated with DPPH solution for 5 min at 25 °C and the absorbance was measured using spectrophotometer at 517 nm. The antioxidant activity was expressed as DPPH˙ scavenging percentage (%). The total phenolic content of the ethanol extracts of seedlings was determined with the Folin-Ciocalteu reagent^67^ and the absorbance of solutions was determined at 765 nm with the spectrophotometer. The quantitative value of polyphenols was determined as gallic acid equivalents as standard. Flavonoids were extracted and determined according to Zhuang et al.^68^ and the absorbance was measured at 510 nm against the blank. Total flavonoid content was expressed as micrograms of quercetin equivalent per gram of fresh seedling. Carotenoids were determined according to AOAC^69^. Samples were extracted by acetone followed by petroleum ether and measured spectrophotometrically at 450 nm. Ascorbic acid was extracted by 4% TCA (trichloroacetic acid) and DNPH (Dinitrophenyl hydrazine) reagent and thiourea by the method described by Kapur et al.^70^. The absorbance was measured at 540 nm against blank. All the solutions and chemicals used in the analysis were obtained from Sigma-Aldrich and were prepared fresh. Three replicates were used for each measurement.

## Data Availability

The datasets generated and/or analyzed during the current study are not publicly available due to its proprietary nature but are available from the corresponding author on reasonable request.
